# Acute nicotine induces anxiety and disrupts temporal pattern organization of rat exploratory behavior in hole-board: a potential role for the lateral habenula

**DOI:** 10.3389/fncel.2015.00197

**Published:** 2015-06-01

**Authors:** Maurizio Casarrubea, Caitlin Davies, Fabiana Faulisi, Massimo Pierucci, Roberto Colangeli, Lucy Partridge, Stephanie Chambers, Daniel Cassar, Mario Valentino, Richard Muscat, Arcangelo Benigno, Giuseppe Crescimanno, Giuseppe Di Giovanni

**Affiliations:** ^1^Laboratory of Behavioral Physiology, Department of Experimental Biomedicine and Clinical Neurosciences, Human Physiology Section “Giuseppe Pagano”, University of PalermoPalermo, Italy; ^2^Faculty of Medicine and Surgery, Department of Physiology and Biochemistry, University of MaltaMsida, Malta; ^3^School of Biosciences, Cardiff UniversityCardiff, UK

**Keywords:** anxiety, serotonin, dopamine, lateral habenula, nicotine, T-pattern analysis

## Abstract

Nicotine is one of the most addictive drugs of abuse. Tobacco smoking is a major cause of many health problems, and is the first preventable cause of death worldwide. Several findings show that nicotine exerts significant aversive as well as the well-known rewarding motivational effects. Less certain is the anatomical substrate that mediates or enables nicotine aversion. Here, we show that acute nicotine induces anxiogenic-like effects in rats at the doses investigated (0.1, 0.5, and 1.0 mg/kg, i.p.), as measured by the hole-board apparatus and manifested in behaviors such as decreased rearing and head-dipping and increased grooming. No changes in locomotor behavior were observed at any of the nicotine doses given. T-pattern analysis of the behavioral outcomes revealed a drastic reduction and disruption of complex behavioral patterns induced by all three nicotine doses, with the maximum effect for 1 mg/kg. Lesion of the lateral habenula (LHb) induced hyperlocomotion and, strikingly, reversed the nicotine-induced anxiety obtained at 1 mg/kg to an anxiolytic-like effect, as shown by T-pattern analysis. We suggest that the LHb is critically involved in emotional behavior states and in nicotine-induced anxiety, most likely through modulation of monoaminergic nuclei.

## Introduction

Tobacco smoking is a serious health problem worldwide and is well known to be one of the major causes of death in developed countries (Vella and Di Giovanni, 2013). The reinforcing properties of nicotine are thought to be due to increased dopamine (DA) release in the mesolimbic DA system (Corrigall et al., 1992; Di Chiara, 2000). Nicotine exerts its action by binding to nicotinic acetylcholine receptors (nAChRs), which are heterogenous, pentameric channels constructed from multiple combinations of six α (2–7) and three β (2–4) subunits. Besides DA, nAChRs mediate the release of a wide range of neurotransmitters within the central nervous system (CNS), including serotonin (5-HT), *gamma-*aminobutyric acid (GABA), glutamate (GLU) and nitric oxide (Pierucci et al., 2004, 2014; Di Matteo et al., 2010; Di Giovanni, 2012; Lester, 2014).

In addition to its rewarding effects, nicotine is also highly noxious (Fowler and Kenny, 2014). As far as the relationship between smoking and anxiety is concerned, it is one of complex nature as both anxiogenic and anxiolytic nicotinic effects have been described (Picciotto et al., 2002). A common assumption is that cigarette smoking relieves feelings of stress and anxiety, and therefore sustains the addiction. Nevertheless, a growing body of evidence suggests an opposing scenario, in which nicotine is preferentially associated with heightened stress in smokers. Indeed, initial aversion to nicotine experienced by first-time smokers, including anxiety, is a common experience (Newhouse et al., 1990) which subsequently can be a very important factor since it can decrease the likelihood of developing a tobacco addiction (Sartor et al., 2010). Additionally, quitting smoking has been associated with a moderate reduction in anxiety levels at 6 months (McDermott et al., 2013). The rewarding and aversive nicotine effects are likely mediated by a heterogeneous population of nAChR subtypes in different neuronal circuits. The aversive nicotine effects, including anxiety, might be mediated by the lateral habenula (LHb; Fowler and Kenny, 2014), a small epithalamic structure that has been shown to convey negative motivational signals (Bianco and Wilson, 2009).

The LHb has recently attracted significant attention in nicotine action. Several experimental studies indicate that nicotine may influence the DAergic, GABAergic and serotonergic systems (Pierucci et al., 2011; Lecca et al., 2014), whilst also directly activating habenular neurons (Pierucci et al., 2011; Dao et al., 2014; Velasquez et al., 2014), probably via α3α5β4-containing nAChRs highly expressed therein (Salas et al., 2009). Moreover, habenular β4* receptors have been shown to be necessary for nicotine intake and withdrawal symptoms (Salas et al., 2009; Fowler et al., 2011). Finally, the LHb is also involved in nicotine seeking, given that D3 antagonism within this area decreases cue-induced nicotine reinstatement (Khaled et al., 2014). Aside from its role in nicotine and general drug addiction, the LHb is also important for the regulation of behavior and the pathogenesis of several psychiatric disorders, such as depression and schizophrenia (Cui et al., 2014; Lecca et al., 2014).

The LHb control of behavior is complex and the majority of evidence focuses on motivated/punishment behavior. The LHb is likely to be essential for survival through the promotion of learning and subsequent activities that lead to avoidance of stimuli associated with negative consequences. For instance, optogenetic activation of the LHb promotes active and passive avoidance behavior in mice (Stamatakis and Stuber, 2012), while bilateral lesion of the LHb reduces escape and avoidance latencies in rats (Pobbe and Zangrossi, 2010). In addition, under stressful conditions (i.e., induced by yohimbine) the anxiogenic-like response in rats diminishes following inactivation of the LHb (Gill et al., 2013). Moreover, the LHb mediates the aversive effects of alcohol in suppressing voluntary ethanol consumption (Haack et al., 2014). Hence, the LHb might also be a key area of interest in nicotine-induced anxiety, although this hypothesis has not been investigated to date.

The aim of our study is threefold: firstly, to clarify the effect of a wide range of nicotine doses on the anxiety state of animals in the unfamiliar hole-board environment; secondly, to explore the effects of the LHb lesion in comparison to the sham lesion on basal animal emotional reactivity and finally, to evaluate the effect of the LHb lesion on nicotine-induced changes of rat exploratory behavior. We have chosen the hole-board since it is a proven measure to test anxiety state in rodents (Boissier and Simon, 1962; File and Wardill, 1975), and is a useful tool in understanding the effects of a drug in an aversive situation. In this investigation we studied the role of the LHb in exploratory behavior, head-dipping behavior primarily, but also encompassing motor activities such as walking, rearing and grooming (Takeda et al., 1998). Furthermore, we used both quantitative and multivariate T-pattern analysis (for a recent review, see Casarrubea et al., 2015) to evaluate the activity of the unlesioned, sham-lesioned and LHb-lesioned rats in the hole-board, under basal conditions and after nicotine administration. Particularly, T-pattern analysis has been shown to represent a useful tool to detect even small induced behavioral changes (Casarrubea et al., 2010) and evaluate and compare different classes of anti-anxiety molecules (Casarrubea et al., 2011). Here, we show that nicotine induces anxiety-like changes in the animal behavior. Successively, we confirm that the LHb lesion induces hyperactivity and we show, for the first time, it reduces anxiety state and emotionality. We found that the selective bilateral electrolytic lesion of the LHb strikingly reverts the anxiogenic effect of 1 mg/kg of nicotine, as shown by head-dipping behavior. Furthermore, T-pattern analysis showed that in LHb-lesioned animals nicotine-induced anxiolysis is strongly potentiated. Consequently, our results indicate that the LHb is an important area of the anxiety circuitry.

## Materials and Methods

### Animals

Eighty male Sprague-Dawley rats (Charles River, Margate, UK) weighing between 250–350g were used in the HB experiments. Rats were housed in a room kept at a constant temperature of 21 ± 1°C, a relative humidity of 60 ± 5% and under a light: dark cycle of 12 h: 12 h with the lights being turned on at 6 am. Food and water was provided to the animals *ad libitum*. Procedures involving animals and their care were conducted in conformity with European Law and the institutional guidelines, approved by the University of Malta, Faculty of Medicine and Surgery, Animal Welfare Committee. All efforts were made to minimize animal suffering and to reduce the number of animals used.

### Hole-Board

The hole-board apparatus consisted of an open field made up of four 50 cm^2^ Plexiglas walls and a floor divided into 9 equal squares (each 16.6 cm^2^). Three walls had polystyrene attached to them to make them opaque. The fourth wall was clear Plexiglas in order to record the animals via a video camera mounted on a tripod placed a short distance from the HB. The walls were attached to a floor raised 5 cm above the ground surface. The raised floor had four holes, each 4 cm in diameter and positioned equidistant from one another. Additionally, to ensure that each hole was equidistant from their adjacent corners, they were drilled 10 cm from their two neighboring walls (Casarrubea et al., 2009b).

### Lesioning Procedure

**Figure 1:**
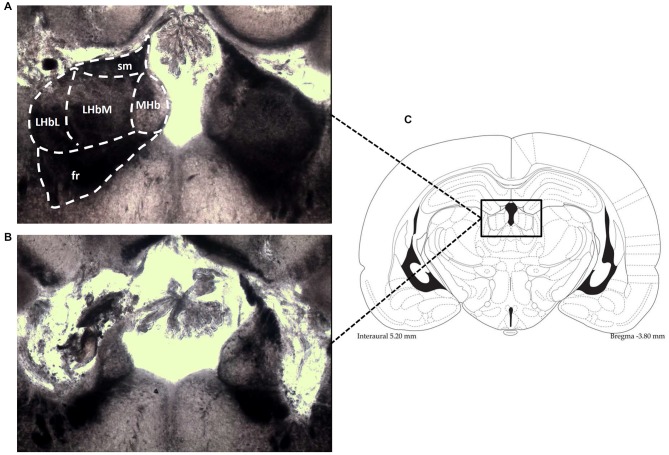
**Photomicrographs (an enlargement of the box in C) demonstrate the typical location of lesions in the LHb (B), shown in contrast to the intact LHb in an unlesioned animal (A)**. Also shown is the corresponding diagramatic representation of the analogous coronal section **(C)**. fr, fasciculus retroflexus; LHbM, Medial part of Lateral Habenula; LHbL, Lateral part of Lateral Habenula; MHb, Medial Habenula; sm, stria medullaris.

Twenty rats received bilateral electrolytic lesions at the LHb level. Two holes were made in the skull, 3.6 mm posterior to bregma and 1.8 mm lateral to the midline (Paxinos and Watson, 2007). Two bipolar electrodes made from two stainless steel bifilar wires (California Fine Wire, Grover Beach, CA, USA) with their ends separated 0.5 mm, were attached to a micromanipulator angled 10° to the coronal plane, and lowered into the right and left LHb (depth of 5.0 mm from the surface of the dura). A 500 μA current was applied for 30 s using an optically isolated stimulator (DS3 Digitimer, Hertfordshire, UK). The electrodes were left in place for a few minutes before removing. The rat was then left to recover from the anesthesia for approximately 1–2 h. Once surgery was complete, rats were given a subcutaneous injection of saline (1 ml) and a topical application of antibiotic cream (mupirocin), and were left for 7–10 days to recover before testing in the hole-board. An identical procedure was followed for twenty additional rats, except electrodes were only lowered −3.5 mm and no current was passed so that no electrolytic lesion was made, producing sham-lesioned animals. The animals were killed at the end of the experiments by decapitation and the brains were removed. To histologically verify the extent of the lesion, the brains were freeze-sectioned in a cryostat. Slices (25 μm) were taken through the entire habenula and mounted on slides. Lesions of the LHb were considered acceptable when surrounding regions (i.e., medial habenula, dorsal hippocampus and thalamic nuclei) were spared (Figure 1).

### Drugs and Treatments

As to unlesioned subjects, the treatment groups were: saline (vehicle), nicotine 0.1 mg/kg, 0.5 mg/kg and 1 mg/kg, all administered intraperitoneally (i.p.). Sham-lesioned and LHb-lesioned rats were treated with saline or nicotine 1 mg/kg, i.p. (−)-Nicotine hydrogen tartrate salt was diluted in saline and adjusted to pH 7.4. All drug doses refer to the weight of the salt. Saline or nicotine was given in 1 ml/kg volume, 30 min before the test.

### Procedure

All recordings took place between 9 am and 1 pm and none of the rats had previously been exposed to the hole-board before experimentation. Each rat received the drug treatment as described previously and was brought into the testing room and left for 30 min to acclimatize. The animals were subsequently placed in the center of the hole-board and allowed to freely explore for 10 min, whilst being recorded by video camera. After each recording the hole-board was cleaned with ethanol (70%) to remove all scent traces and faeces. The video recordings were blind analyzed off-line.

### Data Analysis

**Figure 2:**
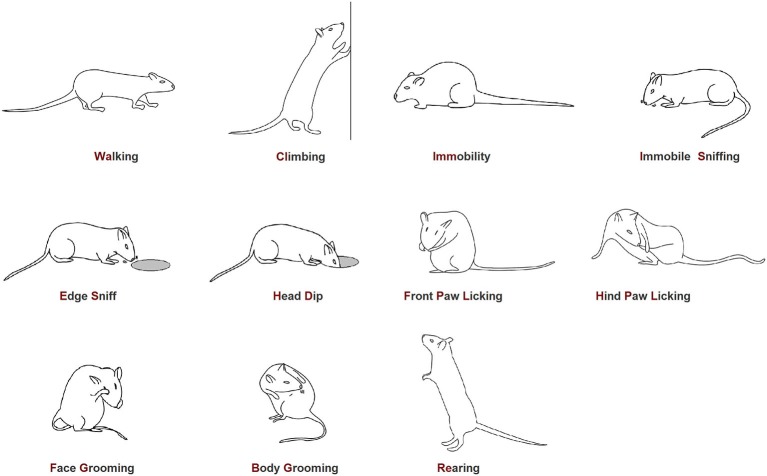
**Ethogram of rat behavior in the hole-board apparatus**. Walking (Wa): the rat walks around sniffing the environment; Climbing (Cl): the rat maintains an erect posture leaning against the Plexiglas wall. Usually associated with sniffing; Immobility (Imm): the rat maintains a fixed posture. No movements are produced; Immobile Sniffing (IS): the rat sniffs the environment standing on the ground; Edge Sniff (ES): the rat sniffs the hole border without inserting the head inside; Head-Dip (HD): the rat puts its head into one of the four holes; Front Paw Licking (FPL): the rat licks or grooms its forepaws; Hind Paw Licking (HPL): the rat licks or grooms its hind paws; Face Grooming (FG): the rat rubs its face (ears, mouth, vibrissae, and eyes) with rapid circular movements of its forepaws; Body Grooming (BG): the rat licks its body combing the fur by fast movements of incisors; Rearing (Re): the rat maintains an erect posture without leaning against the Plexiglas box; usually associated with sniffing.

The ethogram utilized in the present investigation (Figure 2) is the same that we employed in our previous studies (Casarrubea et al., 2009b,c, 2010, 2011). Video files were coded by means of a software coder (*The Observer*, Noldus Information Technology bv, The Netherlands) and event log files generated for each subject. To detect temporal relationships among behavioral elements, event log files were processed with *Theme software* (PatternVision Ltd, Iceland; Noldus Information Technology, The Netherlands). *Theme* is a specific software able to detect repeated sequences of events on the basis of statistically significant constraints on the intervals separating them (Magnusson, 2000). In brief, an algorithm compares the distributions of each pair of the behavioral elements A and B searching for a time window so that, more often than expected by chance, A is followed by B within that time window. In this case, a statistically significant relationships exists between A and B and are, by definition, a T-pattern indicated as (A B). Then, such first level T-patterns are considered as potential A or B terms in higher order patterns, e.g., ((A B) C). And so on, up to any level. A more detailed description of concepts, theories and procedures behind T-pattern analysis can be found in our previous articles (Casarrubea et al., 2009a, 2010, 2011, 2013a,b, 2014, 2015).

The following parameters of the behavioral response were analyzed: (1) mean duration of each behavioral element, for each subject; (2) mean occurrence of each behavioral element, for each subject; (3) overall number of different T-patterns detected for each group both in real and random generated data; (4) structure of all the different T-patterns detected for each group (strings); (5) overall occurrences; and (6) percentage distribution of T-patterns including behaviors of hole-exploration, namely edge-sniffing and head-dipping.

### Statistics

One-way ANOVA, followed by Newman-Keuls *post hoc* test for multiple comparisons, was carried out to assess possible drug-induced modifications of the mean occurrences and mean durations of behavioral elements in saline and nicotine (0.1, 0.5, and 1 mg/kg) administered unlesioned groups.

Two-way ANOVA (treatment × lesion) was used to analyze differences among saline in sham-lesioned rats, saline in LHb-lesioned rats, nicotine 1 mg/kg in sham-lesioned rats and nicotine 1 mg/kg in LHb-lesioned rats, with *post hoc* Fisher’s PLSD test to assess individual group comparisons on most behavioral variables. In the case of a significant effect of lesion group or a significant lesion × treatment interaction, the data of the sham-lesioned and LHb-lesioned groups, comparisons of nicotine to the vehicle control condition were made by paired *t*-tests. Differences were considered significant at *p* < 0.05.

Concerning T-pattern analysis, albeit all detected T-patterns imply a statistical significance among critical intervals separating their events, the enormous amount of possible relationships raises the question of whether the number of different detected T-patterns is different by chance. The software used for T-pattern detection deals with such a crucial issue by repeatedly randomizing and analyzing the original data. In brief, for each group, the mean number of T-patterns + 1 SD detected in random generated data is compared with the actual number of T-patterns detected in real data. Two-way ANOVA (lesion × treatment) was used to analyze differences among saline in sham-lesioned rats, saline in LHb-lesioned rats, nicotine 1 mg/kg i.p. in sham-lesioned rats and nicotine 1 mg/kg i.p. in LHb-lesioned rats. Finally, chi-square test was carried out to compare possible significant differences in the percent distribution of T-patterns.

## Results

### Effects of Saline and Acute Nicotine Administration on Different Behavioral Components of Unlesioned Rats in Hole-Board

**Figure 3:**
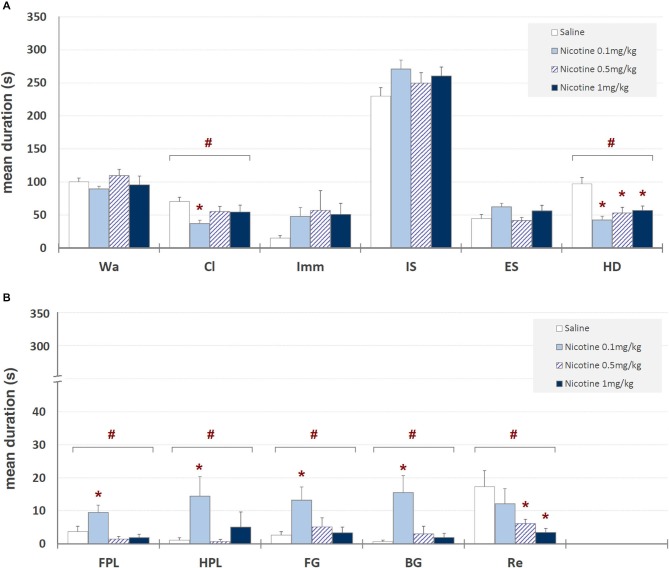
**Mean durations ± SEM (in seconds) of behavioral components in unlesioned groups (saline, nicotine 0.1, 0.5, and 1 mg/kg)**. # = significant (*p* < 0.05) ANOVA result; * = significant (*p* < 0.05) difference in comparison with saline (Newman-Keuls *post hoc* test for multiple comparisons). Panel **(A)** = behavioral components with durations ± SEM in one or more groups > 5 s; panel **(B)** = behavioral components with durations ± SEM in one or more groups < 5 s. See Figure 2 for abbreviations.

Mean durations ± SEM of each behavioral component in saline and nicotine (0.1, 0.5, and 1 mg/kg, i.p.) treated unlesioned groups are presented in Figure 3. One-way ANOVA revealed significant nicotine-related changes for climbing (*F*_3,39_ = 3.19, *p* < 0.035), head-dipping (*F*_3,39_ = 9.58, *p* < 0.0001), front paw licking (*F*_3,39_ = 6.07, *p* < 0.002), hind paw licking (*F*_3,39_ = 2.87, *p* < 0.05), face grooming (*F*_3,39_ = 3.58, *p* < 0.023), body grooming (*F*_3,39_ = 5.69, *p* < 0.003) and rearing (*F*_3,39_ = 3.28, *p* < 0.032). Newman-Keuls *post hoc* test showed significant (*p* < 0.05) nicotine-induced decreases, in comparison with saline, for head-dipping at all nicotine doses, for rearing at 0.5 and 1 mg/kg and for climbing at 0.1 mg/kg, while a significant increase was observed for front paw licking, hind paw licking, face grooming and body grooming at 0.1 mg/kg.

**Figure 4:**
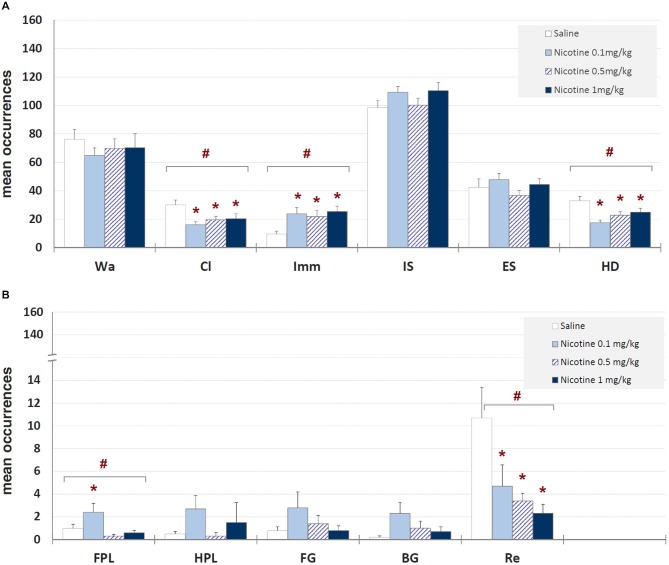
**Mean occurrences ± SEM of behavioral components in unlesioned groups (saline, nicotine 0.1, 0.5, and 1 mg/kg)**. # = significant (*p* < 0.05) ANOVA result; * = significant (*p* < 0.05) difference in comparison with saline (Newman-Keuls *post hoc* test for multiple comparisons). Panel **(A)** = behavioral components with occurrences ± SEM in one or more groups > 5; panel **(B)** = behavioral components with occurrences ± SEM in one or more groups < 5. See Figure 2 for abbreviations.

Mean occurrences ± SEM of each behavioral component in saline and nicotine (0.1, 0.5, and 1 mg/kg) injected groups are illustrated in Figure 4. One-way ANOVA showed significant drug-related changes for climbing (*F*_3,39_ = 4.23, *p* < 0.012), immobility (*F*_3,39_ = 3.72, *p* < 0.020), head-dipping (*F*_3,39_ = 6.53, *p* < 0.001), front paw licking (*F*_3,39_ = 4.23, *p* < 0.012) and rearing (*F*_3,39_ = 3.61, *p* < 0.022). Newman-Keuls *post hoc* test highlighted significant (*p* < 0.05) decreases, in comparison with saline, for climbing, rearing and head-dipping at all doses, and an increase of immobility and front paw licking at all nicotine doses and 0.1 mg/kg, respectively. These findings clearly show an anxiogenic-like effect of all the doses of nicotine tested.

### Effects of Saline and Acute Nicotine Administration on T-Pattern Analysis of the Different Behavioral Structure in Unlesioned Rats

**Figure 5:**
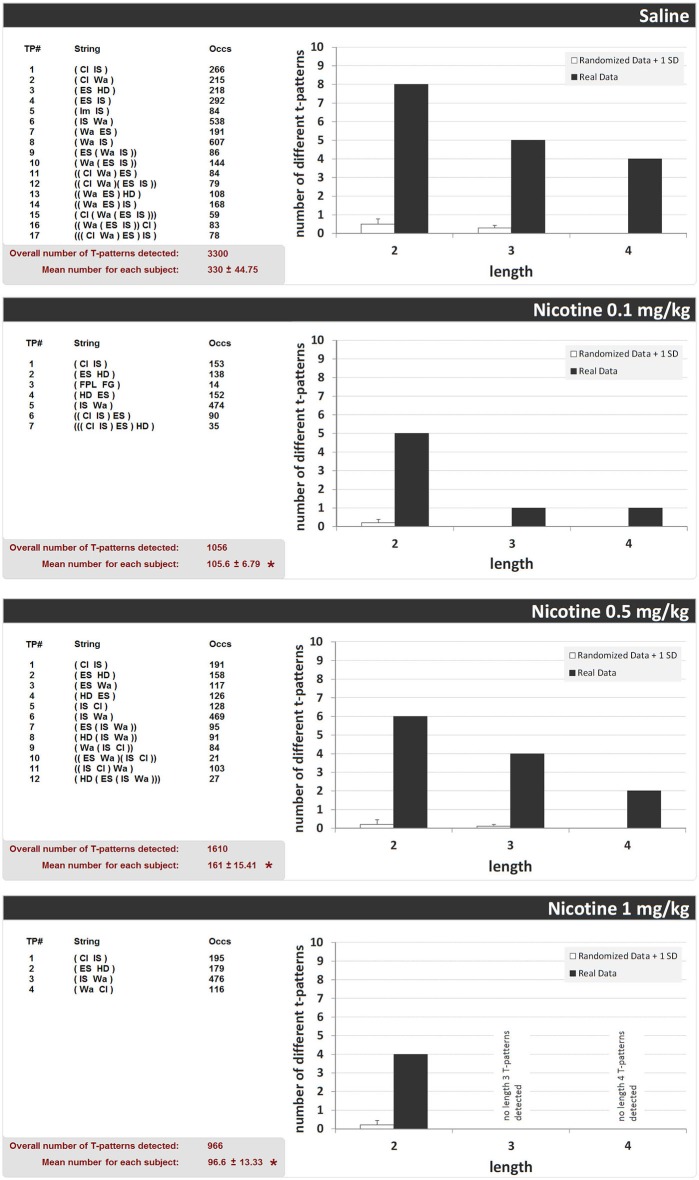
**T-patterns detected in unlesioned groups (saline, nicotine 0.1, 0.5, and 1 mg/kg)**. “TP#” column: number of each different T-pattern detected; “String” column: events encompassed in T-pattern’s structure; “Occs” column: occurrences of each T-pattern. Histograms: T-patterns length distribution in real data (dark bars) and random generated data + 1SD (white bars). Bottom left of each panel: overall T-patterns detected in the group and mean number of T-patterns for each subject. * = significant difference in comparison with saline (ANOVA + Newman-Keuls *post hoc* test for multiple comparisons). See Figure 2 for abbreviations.

Figure 5 shows the structure of all T-patterns detected in unlesioned rats treated with saline or nicotine (0.1, 0.5, and 1 mg/kg, i.p.). For each T-pattern, its terminal string (i.e., events in T-pattern’s structural sequence) and occurrences are indicated. 17 different T-patterns were detected in the saline-administered group. Nicotine 0.1, 0.5, and 1 mg/kg groups revealed 7, 12 and 4 different T-patterns, respectively. Figure 5 also shows, for each group, T-pattern length distribution in real data and in randomly generated data ± 1 SD. For all groups, T-patterns search run performed on random vs. real data demonstrated that the largest amount of different T-patterns detected is present, by far, in real data (Figure 5, dark bars) rather than in randomly generated data (Figure 5, white bars). Finally, the mean number of T-patterns shows a clear-cut reduction in all nicotine-administered unlesioned groups (Figure 5, bottom left of each panel). ANOVA (*F*_3,39_ = 19.03, *p* < 0.0001), followed by Newman-Keuls *post hoc* test for multiple comparisons revealed, in comparison with saline, significant reductions of T-patterns in all nicotine administered groups. More T-patterns arise, by far, in real data than in randomized data for all doses of nicotine, suggesting firstly that the outcome number of T-patterns for all treatments was not due to chance (Casarrubea et al., 2011).

### Effects of Bilateral LHb Lesion on Saline and Nicotine-Induced Changes of Different Behavioral Components in Hole-Board

Of the 20 rats that underwent LHb lesion, four in both the saline and nicotine groups did not have a satisfactory lesion, and so were not included in the statistical analysis. Otherwise, no data were excluded from analysis. Of the 20 rats that underwent sham lesioning, 3 in both saline and nicotine group were excluded for complications relating to the operation.

**Figure 6:**
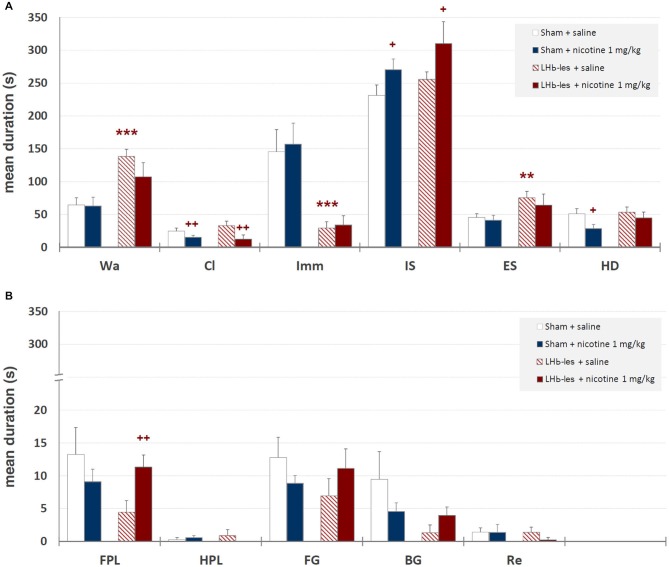
**Mean durations ± SEM of behavioral components in sham-lesioned groups (sham + saline and sham + nicotine 1 mg/kg) and LHb-lesioned groups (LHb-les + saline and LHb-les + nicotine 1 mg/kg)**. *= *p* < 0.05 compared to the sham-lesioned group after the same drug treatment; ** = *p* < 0.01 compared to the sham-lesioned group after the same drug treatment; *** = *p* < 0.005 compared to the sham-lesioned group after the same drug treatment; + = *p* < 0.05 compared to the same group saline condition (two-tailed paired *t*-test). Panel **(A)** = behavioral components with durations ± SEM in one or more groups > 5 s; panel **(B)** = behavioral components with durations ± SEM in one or more groups < 5 s. See Figure 2 for abbreviations.

**Figure 7:**
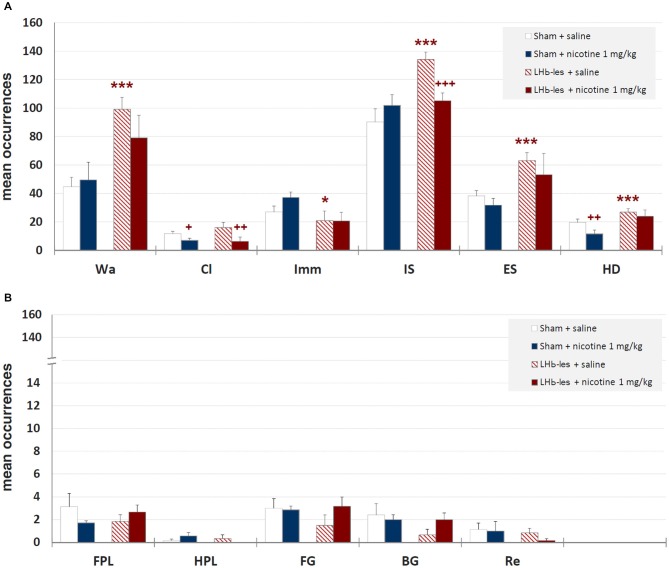
**Mean occurrences ± SEM of behavioral components in sham-lesioned groups (sham + saline and sham + nicotine 1 mg/kg) and LHb-lesioned groups (LHb-les + saline and LHb-les + nicotine 1 mg/kg)**. * = *p* < 0.05 compared to the sham-lesioned group after the same drug treatment; ** = *p* < 0.01 compared to the sham-lesioned group after the same drug treatment; *** = *p* < 0.005 compared to the sham-lesioned group after the same drug treatment; + = *p* < 0.05 compared to the same group saline condition (two-tailed paired *t*-test). Panel **(A)** = behavioral components with occurrences ± SEM in one or more groups > 5; panel **(B)** = behavioral components with occurrences ± SEM in one or more groups < 5. See Figure 2 for abbreviations.

Mean durations ± SEM of each behavioral component are illustrated in Figure 6 while mean occurrences ± SEM of each component are presented in Figure 7. Saline-treated sham-lesioned animals, in comparison to unlesioned animals, exhibit significant changes in different behavioral components for both durations and occurrences (Wa, Cl, HD, FG, BG, and Re; *p* < 0.05) as measured in hole-board, reflecting an enhanced anxiety-like state.

#### Walking

Two-way ANOVA showed significant differences between sham-lesioned and LHb-lesioned groups (*F*_1,22_ = 16.8; *p* = 0.0005), no significant effect of nicotine treatment *F*_1,22_ = 1.3; *p* = 0.28), and a lack of interaction between the two factors (lesion × treatment; *F*_1,22_ = 1.01; *p* = 0.33) on walking mean duration (Figure 6). Similar results were observed for the mean occurrences of walking behavior (lesion *F*_1,22_ = 14.2; *p* = 0.001; treatment *F*_1,22_ = 0.5; *p* = 0.5; lesion × treatment *F*_1,22_ = 1.3; *p* = 0.27; Figure 7).

#### Climbing

Two-way ANOVA revealed a non-significant effect of LHb-lesion (*F*_1,22_ = 0.31; *p* = 0.59) and a significant main effect of treatment (*F*_1,22_ = 8.8; *p* = 0.007) on climbing mean duration. However, no significant interaction of the two factors was observed (*F*_1,22_ = 0.1; *p* = 0.3; Figure 6). Similarly, there was a no significant effect of lesion (*F*_1,22_ = 0.6; *p* = 0.5) and a significant effect of drug treatment (*F*_1,22_ = 8.9; *p* = 0.007) on the occurrence of climbing behavior. In addition, no significant interaction of lesion group × drug treatment (*F*_1,22_ = 1.2; *p* = 0.3) was observed on the occurrence of climbing behavior (Figure 7).

#### Immobility

There was a significant effect of lesion on the duration (*F*_1,22_ = 20.7; *p* = 0.0002) and mean occurrence (*F*_1,22_ = 4.7; *p* = 0.05) of immobility. No significant effect of drug treatment on the duration (*F*_1,22_ = 0.1; *p* = 0.8) and mean occurrence (*F*_1,22_ = 0.9; *p* = 0.4) was observed, whilst there was also no significant interaction of lesion × treatment on the duration (*F*_1,22_ = 0.02; *p* = 0.9) and mean occurrence (*F*_1,22_ = 1.0; *p* = 0.3; Figures 6, 7).

#### Immobile-Sniffing

There was no significant effect of lesion on the duration of immobile sniffing (*F*_1,22_ = 2.2; *p* = 0.1), and a significant effect of drug treatment (*F*_1,22_ = 4.8; *p* = 0.04), but no significant interaction of lesion × drug treatment (*F*_1,22_ = 0.1; *p* = 0.7; Figure 6). As for occurrence, there was a significant effect of lesion (*F*_1,22_ = 10.3; *p* = 0.004), no significant effect of drug treatment (*F*_1,22_ = 1.4; *p* = 0.2), but a significant interaction of these factors (*F*_1,22_ = 7.6; *p* < 0.05) as revealed by two-way ANOVA (Figure 7). *Post hoc* analysis revealed that LHb lesion induced a significant increase in the occurrence (*p* = 0.002) of immobile sniffing in the saline group. Nicotine reduced the mean occurrence (*p* = 0.003) in LHb-lesioned animals and was ineffective in sham-lesioned animals (Figures 6, 7).

#### Edge Sniff

There was a significant effect of lesion on the duration (*F*_1,22_ = 3.8; *p* = 0.01) and on mean occurrence (*F*_1,22_ = 3.6; *p* = 0.008) of edge sniff. Conversely, there was no significant effect of drug treatment on duration (*F*_1,22_ = 0.6; *p* = 0.4) and mean occurrence (*F*_1,22_ = 1.1; *p* = 0.3), nor any significant interaction of lesion × treatment on duration (*F*_1,22_ = 0.1; *p* = 0.7; Figures 6, 7) or frequencies (*F*_1,22_ = 0.05; *p* = 0.8).

#### Head-Dipping

There was no significant effect of lesion on the duration of head-dipping behavior (*F*_1,22_ = 1.5; *p* = 0.2), while a significant effect of drug treatment (*F*_1,22_ = 4.1; *p* = 0.05), but no significant interaction of lesion × treatment (*F*_1,22_ = 0.8; *p* = 0.4) were observed (Figure 6). As for occurrence, there was a strong significant effect of lesion (*F*_1,22_ = 10.8; *p* = 0.003), but no effect of drug treatment (*F*_1,22_ = 3.4; *p* = 0.08) or interaction of these factors (*F*_1,22_ = 0.7; *p* = 0.4; Figure 6).

#### Front Paw Licking

There was no significant effect of lesion on the duration of front paw licking behavior (*F*_1,22_ = 1.5; *p* = 0.2), no significant effect of drug treatment (*F*_1,22_ = 0.2; *p* = 0.6), but significant interaction of lesion × treatment (*F*_1,22_ = 4.1; *p* = 0.05; Figure 6). As for occurrence, there was no significant effect of lesion group (*F*_1,22_ = 0.05; *p* = 0.8), no effect of drug treatment (*F*_1,22_ = 0.5; *p* = 0.7) and no interaction of these factors (*F*_1,22_ = 2.2; *p* = 0.1; Figure 7). *Post hoc* analysis revealed that LHb lesion induced a significant decrease in the duration (*p* = 0.005) of front paw licking. Nicotine did change duration and occurrence in sham-lesioned animals (*p* = 0.3 for both groups), but increased duration in LHb-lesioned rats (*p* = 0.05).

#### Hind Paw Licking

There was no effect of the LHb lesion on the duration (*F*_1,22_ = 0.1; *p* = 0.9) nor on mean occurrence (*F*_1,22_ = 0.6; *p* = 0.4) of hind paw licking. Neither was there a significant effect of drug treatment on duration (*F*_1,22_ = 0.4; *p* = 0.5) and mean occurrence (*F*_1,22_ = 0.1; *p* = 0.8) nor any significant interaction of lesion group × drug treatment for duration (*F*_1,22_ = 1.6; *p* = 0.2) and occurrences (*F*_1,22_ = 2.6; *p* = 0.1; Figures 6, 7).

#### Face Grooming

There was no significant effect of lesion on the duration (*F*_1,22_ = 0.5; *p* = 0.5) or on mean occurrence (*F*_1,22_ = 0.6; *p* = 0.5) of face grooming. Moreover, there was no significant effect of drug treatment on duration (*F*_1,22_ = 0.01; *p* = 0.9) and mean occurrence (*F*_1,22_ = 1.1; *p* = 0.3) nor any significant interaction of lesion group by drug treatment for duration (*F*_1,22_ = 2.5; *p* = 0.1) and occurrences (*F*_1,22_ = 1.4; *p* = 0.2; Figures 6, 7).

##### Body Grooming

There was no significant effect of lesion group on the duration of body grooming behavior (*F*_1,22_ = 3.0; *p* = 0.09), nor significant effect of drug treatment (*F*_1,22_ = 0.2; *p* = 0.7), and neither was there an interaction of lesion × treatment (*F*_1,22_ = 2.2; *p* = 0.1; Figure 6). As for occurrence, there was no significant effect of lesion group (*F*_1,22_ = 1.7; *p* = 0.2), nor significant effect of drug treatment (*F*_1,22_ = 1.7; *p* = 0.2) nor any significant interaction of these factors (*F*_1,22_ = 1.7; *p* = 0.2; Figure 7).

##### Rearing

There was no effect of lesion on the duration (*F*_1,22_ = 0.5; *p* = 0.5) nor on mean occurrence (*F*_1,22_ = 1.0; *p* = 0.34) of rearing. Neither was there a significant effect of drug treatment on duration (*F*_1,22_ = 0.5; *p* = 0.5) and mean occurrence (*F*_1,22_ = 0.2; *p* = 0.7), nor any significant interaction of lesion × drug treatment for duration (*F*_1,22_ = 0.5; *p* = 0.5) and frequency (*F*_1,22_ = 0.2; *p* = 0.7; Figures 6, 7).

### Effects of Bilateral LHb Lesion on T-Pattern Analysis of Saline and Nicotine-Induced Different Behavioral Components in Hole-Board

**Figure 8:**
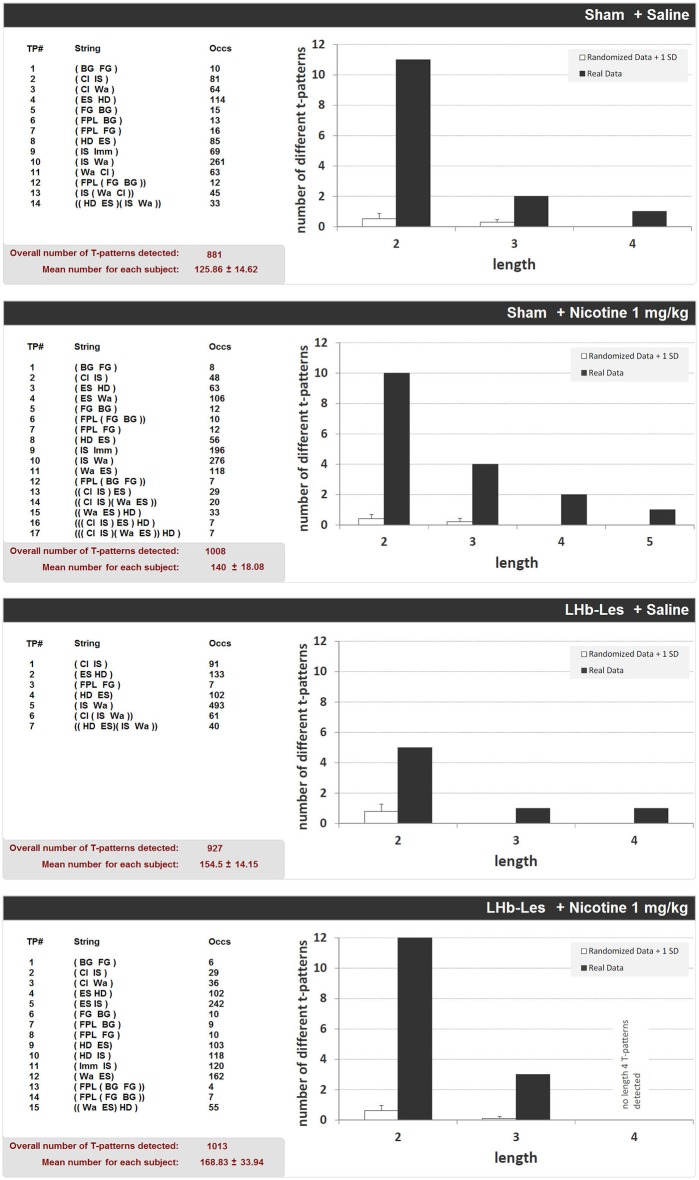
**T-patterns detected in sham-lesioned groups (sham + saline and sham + nicotine 1 mg/kg) and in LHb-lesioned groups (LHb-les + saline and LHb-les + nicotine 1 mg/kg)**. “TP#” column: number of each different T-pattern detected; “String” column: events encompassed in T-pattern’s structure; “Occs” column: occurrences of each T-pattern. Histograms: T-patterns length distribution in real data (dark bars) and random generated data + 1 SD (white bars). Bottom left of each panel: overall T-patterns detected in the group and mean number of T-patterns for each subject. See Figure 2 for abbreviations.

Figure 8 shows the structure of all T-patterns detected in sham-lesioned and LHb-lesioned subjects injected with saline or 1 mg/kg nicotine. In the same way as Figure 5, for each T-pattern, its terminal string and occurrences are indicated. 14 different T-patterns have been detected in sham-lesioned + saline group; 17 in sham-lesioned + nicotine 1 mg/kg; 7 different T-patterns have been detected in LHb-lesioned + saline administered group; 15 different T-patterns have been found in nicotine 1 mg/kg administered group. Both for sham and LHb-lesioned groups, T-patterns search run performed on random vs. real data demonstrated that the largest amount of different T-patterns detected is present, by far, in real data (Figure 8, dark bars) rather than that which is randomly generated (Figure 8, white bars). There was no significant effect of the lesion group on the T-pattern mean occurrence (*F*_1,22_ = 1.6; *p* = 0.2) nor significant effect of drug treatment (*F*_1,22_ = 0.6; *p* = 0.5), or interaction of lesion × treatment (*F*_1,22_ = 0.6; *p* = 0.9; Figure 8).

**Figure 9:**
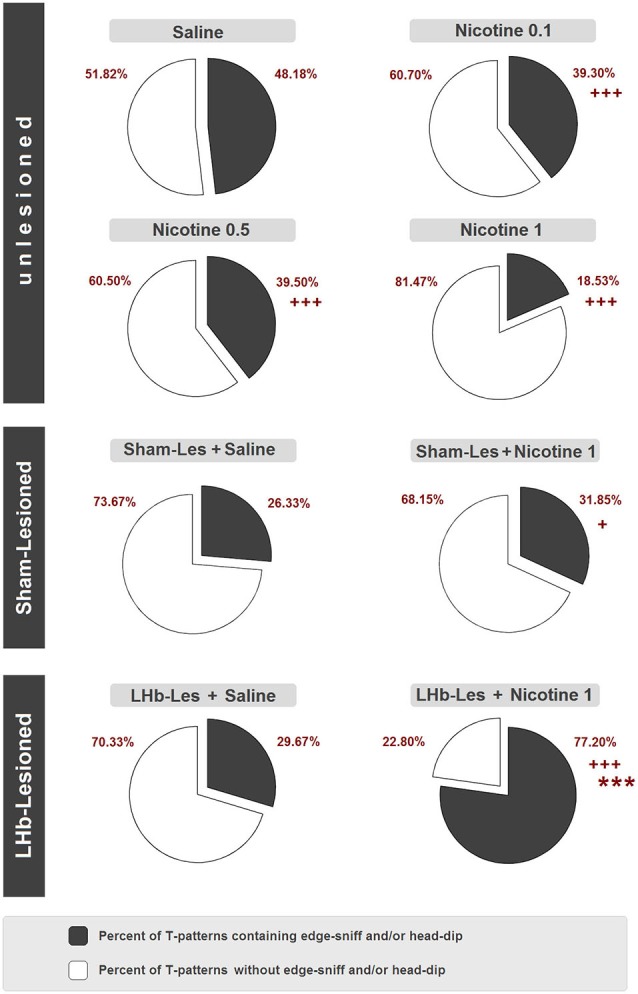
**Percent distribution of T-patterns containing edge-sniff and/or head dip in unlesioned groups (saline, nicotine 0.1, nicotine 0.5, and nicotine 1) sham-lesioned groups (sham-les + saline and sham-les + nicotine 1 mg/kg) and in LHb-lesioned groups (LHb-les + saline, LHb-les + nicotine 1)**. *** = *p* < 0.0001 compared to the sham-lesioned group after the same drug treatment; + = *p* < 0.05, ++ = *p* < 0.005; +++ = *p* < 0.0001 compared to the same group under saline condition (chi-square test).

Finally, Figure 9 illustrates percent distributions of T-patterns containing hole-exploratory behavioral components (i.e., edge sniff and/or head dip) in unlesioned, sham-lesioned and LHb-lesioned groups. Concerning unlesioned animals, in comparison with saline group where 48.2% of T-patterns contained edge sniff and/or head dip, significant (*p* < 0.0001) reductions were detected following nicotine administration at all doses, ranging from 39.3% in nicotine 0.1 mg/kg, to 39.5% in nicotine 0.5 mg/kg, to 18.5% in nicotine 1 mg/kg. With regard to lesioned subjects, there was no significant difference between sham lesion + saline (26.3%) and LHb-lesion + saline (29.7%). On the contrary, the LHb-lesioned + nicotine 1 mg/kg group showed a significant clear-cut increase of T-patterns containing edge sniff and/or head-dip (77.2%), in comparison with the LHb-lesioned saline group (29.7%) (*p* < 0.0001). Concerning sham-lesioned animals, the administration of nicotine induced a lesser but still significant (*p* < 0.05) increase of T-patterns containing edge sniff and/or head-dip, from 26.3% to 31.9%. Finally, highly significant differences (*p* < 0.0001) were also detected between sham-lesioned vs. LHb-lesioned, nicotine 1 mg/kg groups.

## Discussion

The first aim of the current study was to resolve the seemingly conflicting observations in the literature regarding the link between nicotine and anxiety, by directly comparing the effects of different doses of nicotine on anxiety-like animal behavior using hole-board apparatus and quantitative and qualitative analysis. We demonstrated that acute administration of medium-high doses of nicotine (0.1–1 mg/kg, i.p.) induced clear anxiogenic-like effects in normal (unlesioned) rats. Specifically, the hole-board findings showed an anxiogenic-like profile of all doses of nicotine when compared to control, observed 30 min after injection. The total time spent in head-dipping was statistically decreased by nicotine. Strikingly, the more anxiogenic-like nicotine effect was observed at the lower dose, although no statistical difference was identified among different doses. Similarly, all the nicotine doses (0.1–1 mg/kg, i.p.) decreased head-dipping mean occurrence. The duration of rearing was significantly reduced following doses of 0.5 and 1 mg/kg, while climbing was reduced only at 0.1 mg/kg; however, their occurrences were reduced by all the doses compared to control. This effect following acute nicotine treatment is coherent with previous studies, which showed an anxiogenic-like effect following the acute administration of nicotine at 0.25–0.5 (Zarrindast et al., 2000), and 0.5–1.0 mg/kg doses (Ouagazzal et al., 1999a; Hayase, 2007; Zarrindast et al., 2010) in the elevated plus maze (EPM) in rats and mice, and 0.5 mg/kg measured by hole-board in mice (Nasehi et al., 2011). On the other hand, an anxiolytic-like nicotine response has been observed with lower nicotine doses (0.01, 0.05 and 0.1 mg/kg; File et al., 1998; Ouagazzal et al., 1999a; Picciotto et al., 2002; Zarrindast et al., 2010; Varani et al., 2012). Nevertheless, in our conditions, the low dose of 0.1 mg/kg nicotine also induced anxiety-like behavior in rats. In agreement with previous evidence (Zarrindast et al., 2000, 2010; Nasehi et al., 2011), the mean walking duration and occurrence were not significantly different between treatment groups, indicating that the nicotine-induced anxiogenic-like reductions in head-dips and rearing were not due to changes in locomotory activity. Grooming is another useful behavioral parameter to consider, as it is indicative of anxiety levels and is thought to be initiated in response to changes occurring in the animal as a result of anxiogenic stimuli (Spruijt et al., 1992; Kalueff and Tuohimaa, 2005). Consistent with the changes observed on head-dipping and rearing, grooming duration for the different behavioral components (FPL, HPL, FG, and BG) also appeared to be significantly increased by nicotine treatments. Furthermore, multivariate T-pattern analysis revealed that the number of different T-patterns, their overall occurrences and their mean number are significantly reduced in all nicotine-administered groups, with a maximum effect observed at the higher 1 mg/kg dose, showing that nicotine strongly affects the complex behavior structure in unlesioned rats, drastically simplifying it. Thus, it is possible to conclude that acute nicotine administration has a dramatic negative impact in terms of behavioral variability and organization. On the other hand, our data suggest that the acute administration of nicotine induces an increase in the anxiety-like level in the normal animal as indicated, for instance, by the consistent reduction of head-dipping duration, an important index of anxiety (Takeda et al., 1998). It could be inferred that the simplification of temporal characteristics of behavior is linked to an increased anxiety condition induced by the acute nicotine administration. However, the simple assessment of T-patterns quantitative features, such as duration and occurrence, is not sufficient to assess whether the animal behavior modifications are coherent with anxiety. To address this, we conducted a subsequent evaluation of the sequential structure of T-patterns detected containing edge-sniffing and head-dip following our previous studies (Casarrubea et al., 2009b,c) and we found that nicotine administration reduced them in a significant and almost dose-dependent fashion (Figure 9). Thus, behavior structure is significantly reorganized in terms of a reduced exploratory approach, consistent with an increased anxiety-like level. Our findings support some epidemiological studies suggesting that nicotine dependence increases the risk of anxiety disorder and panic attacks (Bruijnzeel, 2012). Indeed, first-time smokers report aversion to nicotine and increased anxiety (Newhouse et al., 1990), while long-term smokers show higher levels of anxiety and stress compared to non-smokers (Parrott and Murphy, 2012). In line with this, a moderate reduction in anxiety levels has been observed 6 months after quitting smoking (McDermott et al., 2013).

The contradictory evidence surrounding nicotine and anxiety might be explained by regional nAChR subunit configuration (File et al., 2000). Indeed, α4-nAChR knock out (KO) mice have decreased anxiety-like behavior (Ross et al., 2000; McGranahan et al., 2011), while α7- (Paylor et al., 1998), β3- (Booker et al., 2007) and β4-nAChR KO mice (Salas et al., 2003) seem to present an increase in anxiety-related behavior. Interestingly, elimination of α4β2-nAChRs specifically from DAergic neurons decreases sensitivity to the anxiolytic effects of nicotine (McGranahan et al., 2011). Recently, it has been suggested that low dose nicotine inhibits β2* nAChRs inducing the anxiolytic-like effects, while high doses stimulate them leading to the anxiogenic-like effects of nicotine (Anderson and Brunzell, 2015).

Apart from the different nAChRs in the brain, the complex behavioral output following nicotine administration depends on (i) the different brain areas involved in anxiety as a whole; and (ii) the neurotransmitter systems regulated by nAChRs all taken together. Local administration studies in animals have identified different brain areas that may be involved in the modulation of anxiety by nicotine and endogenous ACh. Bilateral administration of nicotine into the central amygdala (Zarrindast et al., 2008, 2013), the dorsal raphe nucleus (DRN; Cheeta et al., 2001), lateral septal nucleus (Ouagazzal et al., 1999b) and hippocampus (Ouagazzal et al., 1999a; Kenny et al., 2000), or applied to different areas of the mesolimbic DA system (Picciotto et al., 2002; Zarrindast et al., 2013) has been shown to induce an anxiogenic-like effect. Of note, nicotine injection into the DRN has differential effects on behavior in the social interaction test depending on the dose used. Low doses of nicotine are anxiolytic, intermediate doses have no effect, and high doses are anxiogenic (Cheeta et al., 2001). As of yet, no data exist regarding the involvement of the LHb in nicotine-induced anxiety-like behavior in animals.

In the second part of our study we showed a significant change in the locomotor activity in rats in the hole-board after LHb lesion when compared to sham-lesioned rats, as previously observed in many other studies (Nielson and McIver, 1966; Lecourtier et al., 2008; Gifuni et al., 2012; Wang et al., 2013; Jean-Richard Dit Bressel and McNally, 2014) validating the manipulation within the current study. This locomotor effect is likely due to the strong inhibitory control over midbrain DA neurons exerted by the LHb (Matsumoto and Hikosaka, 2007). Moreover, the occurrence, but not the total time of immobile sniffing and head-dipping, were significantly increased in the LHb-lesioned animals, while no changes in the grooming were revealed, suggesting an anxiolytic effect of the removal of the LHb influence. Strikingly, 1.0 mg/kg nicotine in LHb-lesioned animals was unable to produce the same anxiogenic effects (as change of head-dipping occurrence and duration) compared to 1.0 mg/kg acute nicotine treatment in sham-lesioned animals. While climbing was further inhibited, grooming was increased by nicotine in LHb-lesioned animals (although not significantly). Interestingly, the LHb lesion changed the direction of nicotine effect on immobile sniffing, decreasing it compared to the LHb-lesioned animals that receive saline.

Concerning T-pattern analysis, sham-lesioned and LHb-lesioned rats treated with saline are characterized by a modification of anxiety-related behavior compared to unlesioned animals. Indeed, strings (Figure 8) and percentage of T-patterns containing edge-sniff and/or head-dipping (Figure 9) describe, in both sham and LHb-lesioned animals, a situation essentially consistent with an increased anxiety level, although the influence of the hypolocomotion induced by surgery cannot be excluded. The above discussed condition of increased anxiety, in rats with lesion of the LHb, radically changes if nicotine is acutely administered. Figures 8, 9 clearly demonstrate that following nicotine administration in LHb-lesioned rats, the number of T-patterns containing head-dip and edge sniff is strongly increased. Interestingly, although less evident, nicotine induced an increase in T-patterns containing head-dip and edge sniff in sham-lesioned animals, about 32% compared to the 26% of the saline. LHb-lesioned rats treated with nicotine presented the largest extent of patterns, about 77%, containing edge sniff and head dip. It therefore appears that acute nicotine injected animals with lesion in the LHb do explore the holes significantly more.

Although sham-lesioned animals were in good health (7–10 day recovery), they displayed more anxious behavior than unlesioned rats. Such an outcome demonstrates that the lesioning itself had an evident impact in terms of behavioral organization, as indicated by a decrease in locomotion, rearing and head-dipping and increases in immobility and T-patterns containing head dip and edge sniff; typical of an anxiogenic-like phenotype. Some aspects of the surgical procedure used in this study may have been stressful and it is well known that stress induces anxiogenic-like behavior (Bondi et al., 2008). Thus, some of the nicotine’s anxiolytic activity in sham and LHb-lesioned animals may be related to the drug’s known anxiolytic properties under conditions of stress (Hsu et al., 2007). Strikingly, the LHb lesion strongly amplified the anxiolytic nicotine effect. Such evidence is suggestive of the important role of the LHb in the behavioral organization of the animal following pharmacological modulation (i.e., nicotine) of its emotional reactivity (i.e., anxiety) and in behavioral response to stress.

One of the most important findings of our study is the evidence that standard quantitative analyses (such as duration and occurrence) provide a reductionist portrait of animal behavior. This owes to these approaches describing the behavior in terms of individual components, separate from the comprehensive behavioral architecture. On the other hand, our results using a multivariate approach providing information concerning the structural relationships among each component of the rat behavioral repertoire, show that T-pattern analysis is capable of revealing effects that otherwise would have been neglected, i.e., anxiolytic nicotine activity in LHb-lesioned rats. The case of head-dip duration is explicative; nicotine in LHb-lesioned rats does not affect the duration or occurrences of head-dip compared to its vehicle. As we have discussed in the preceding section, this would have been a wrong conclusion. In reality, when the relationships of head-dip with the other components of the behavior are analyzed, a completely different scenario emerges. The number of head-dips and edge sniffs become components of the largest amount of behavioral sequences performed by the LHb-lesioned animals following nicotine administration. In these animals, the environmental exploration becomes significantly more organized in comparison with the saline administered groups.

Our observations are consistent with evidence that chemical inactivation of the LHb limits and abolishes certain behaviors shown under highlighted anxiety states, such as increasing the time spent in the open arms of the EPM, decreasing the time spent burying in the defensive burying task following yohimbine administration and blunting cocaine seeking that is exacerbated by yohimbine (Gill et al., 2013). Consistently, bilateral electrolytic lesion of the LHb impairs inhibitory avoidance acquisition in the EPM, indicating an anxiolytic-like effect (Pobbe and Zangrossi, 2008). Our data are in agreement with previous findings, which show that lesioning of the fasciculus retroflexus improves the behavioral response of depressed rats by increasing the 5-HT level in the DRN (Yang et al., 2008). Our current findings support and extend these prior studies by showing that the inactivation of the LHb *per se* decreases anxiety-like traits in rats (i.e., increase in head-dipping), an effect never observed before.

However, our data do not allow us to be conclusive about the role of the LHb in general and nicotine-induced anxiety-like behavior. Further studies utilizing larger sample size, multiple behavioral tests and anxiolytic drugs should be conducted to validate our results.

Concurrently, different types of LHb inactivation/lesion, which might potentially produce control animals with lower levels of basal anxiety compared to those used in our current study, should be considered. Our study therefore highlights an important methodological issue when evaluating behavioral studies that are based on comparisons of only lesioned animals with sham-lesioned with no inclusion of unlesioned controls, which form the majority of the available data.

It still remains to be explained how a lesion in this small epithalamic formation reverts acute nicotine-induced anxiety-like behavior. From an anatomical perspective, the LHb, through the stria medullaris, receives inputs mainly from the basal ganglia and from the limbic system (Hikosaka et al., 2008). The output, through the fasciculus retroflexus, is directed to brain structures containing dopaminergic neurons (e.g., substantia nigra pars compacta, VTA) and serotonergic neurons (e.g., DRN, medial raphe nucleus); also, indirect connections take place through the GABA-ergic rostromedial tegmental nucleus (RMTg; Hikosaka, 2010; Proulx et al., 2014). Thus, it is evident that the LHb occupies a key position among pathways involved in the transmission of information concerning emotional processes (limbic input) and motor behavior decision-making processes (basal ganglia input). Indeed, LHb-lesioned rats show for instance a deficit in escape behavior, indicating a role for the habenula in the selection of correct behavioral strategies and innate motor programs (Thornton and Evans, 1982). Thus, the decreased anxiety observed in animals with lesion in the LHb, and the strong anxiolytic-like effects observed following nicotine administration, may depend on the imbalance between DA and 5-HT produced by the disruption of specific bidirectional pathways toward DAergic and serotoninergic systems, both of which are essential in the homeostasis of anxiety/stress levels (Zweifel et al., 2011; Zangrossi and Graeff, 2014).

Specifically, one possible explanation for the present findings is that nicotine, activating the nAChRs located within or outside the LHb, may eventually increase the LHb activity (Pierucci et al., 2011; Dao et al., 2014). This would indirectly cause a reduction in activity of DAergic systems, by strongly increasing the RMTg GABAergic input to the VTA neurons projecting to the lateral shell of the nucleus accumbens (Hong et al., 2011; Lecca et al., 2011; Lammel et al., 2014), decreasing the rewarding effects of nicotine. A direct LHb-VTA excitatory input also exists toward a neuronal subpopulation of the medial VTA that mediates aversion and projects to the medial prefrontal cortex (mPFC; Lammel et al., 2014). The mPFC forms part of the anxiety network and has been shown to modulate the amygdala, bed nucleus of the stria terminalis and ventral hippocampal neuronal activity, synchronizing them on the theta band during high state of anxiety (Adhikari, 2014). Evidence that the LHb spontaneously generates theta oscillations in phase with hippocampus (Goutagny et al., 2013) further suggests that the LHb might also be considered part of the anxiety brain network.

The LHb couples the DA and 5-HT systems, and nicotinic activation of the LHb may modulate 5-HT neuronal activity of the raphe nuclei, directly and indirectly via the RMTg (Sego et al., 2014; Zhao et al., 2015). The LHb-RMTg projection is inhibitory on a DRN subpopulation of presumptive glutamatergic neurons, while the direct LHb-DRN is excitatory on distinctive 5-HT-containing neurons area (Sego et al., 2014). Therefore, nicotine acting on the LHb would increase 5-HT neuronal activity and its release in several brain regions (Pierucci et al., 2014), including mPFC, hippocampus and amygdala leading to the development of an anxiety state. Strikingly, in our conditions the LHb lesion reverses the anxiogenic-like effect mediated by 1 mg/kg of nicotine into an anxiolytic-like effect. The LHb lesion might produce some neurochemical (i.e., DA, 5-HT, glutamate, GABA) or hormonal (e.g., corticosterone) changes which indirectly antagonize the anxiety state induced by nicotine treatment. The nature of such an interaction is far from being simple. Firstly, it is very difficult to tease apart the different contributions of the single LHb projections and the consequences of removing the LHb in modulating nicotine effects. Secondly, nAChRs are highly represented in all the areas of the anxiety network, including DA and 5-HT areas.

Further investigations with habenular lesion/activation, together with measurements of differential neurochemical and behavioral alterations under normal and stressful situations are needed to clarify the nature of the function of the habenular complex in general and nicotine-induced anxiety phenotype.

In conclusion, this study demonstrates that nicotine itself leads to anxiety-like behavior under normal conditions and acts as an anxiolytic under some circumstances (i.e., stressful conditions). The LHb greatly potentiates the anxiolytic-like properties of nicotine, further supporting the role of the LHb in the neuronal circuits that mediates nicotine’s aversive effects (Fowler and Kenny, 2014).

Moreover, from a methodological point of view, an important output of our research is the evidence of the necessity of a synergic use of both quantitative and multivariate analyses to gain a precise description of the effects induced by one or more independent variables in animal behavior analysis.

Nevertheless, much work still remains to be done. Our data support the interesting possibility that increasing the noxious properties of nicotine, acting at the level of the LHb, may serve as a novel strategy for the development of efficacious smoking cessation agents.

## Conflict of Interest Statement

The authors declare that the research was conducted in the absence of any commercial or financial relationships that could be construed as a potential conflict of interest.
